# ExerSense: Physical Exercise Recognition and Counting Algorithm from Wearables Robust to Positioning †

**DOI:** 10.3390/s21010091

**Published:** 2020-12-25

**Authors:** Shun Ishii, Anna Yokokubo, Mika Luimula, Guillaume Lopez

**Affiliations:** 1 Intelligence and Information Course, Aoyama Gakuin University, Sagamihara 252-5258, Japan; sishii@wil-aoyama.jp (S.I.); yokokubo@it.aoyama.ac.jp (A.Y.); 2 ICT Unit, Faculty of Engineering and Business, Turku University of Applied Sciences, 20520 Turku, Finland; mika.luimula@turkuamk.fi

**Keywords:** wearable, sports, human activity recognition, accelerometer

## Abstract

Wearable devices are currently popular for fitness tracking. However, these general usage devices only can track limited and prespecified exercises. In our previous work, we introduced ExerSense that segments, classifies, and counts multiple physical exercises in real-time based on a correlation method. It also can track user-specified exercises collected only one motion in advance. This paper is the extension of that work. We collected acceleration data for five types of regular exercises by four different wearable devices. To find the best accurate device and its position for multiple exercise recognition, we conducted 50 times random validations. Our result shows the robustness of ExerSense, working well with various devices. Among the four general usage devices, the chest-mounted sensor is the best for our target exercises, and the upper-arm-mounted smartphone is a close second. The wrist-mounted smartwatch is third, and the worst one is the ear-mounted sensor.

## 1. Introduction

Exercise and physical activity have well-documented mental and physical health benefits [1,2]. People who partake in regular physical activity are healthier and have a better mood. They are also less prone to several chronic diseases (e.g., cardiovascular disease, diabetes, cancer, hypertension, obesity, and depression) and live much longer compared to those with a sedentary lifestyle. Consequently, active daily living is recommended for all people of all ages [2]. Unfortunately, despite the numerous benefits of regular physical activity, it is challenging for most people to stay motivated and keep adherence to a regular workout schedule [3]. Indeed, people easily lose self-motivation. Additionally, at least for beginners, proper physical exercise necessitates training.

Researchers and exercise therapists have proposed numerous strategies that help improve adherence to a regular exercise schedule [4]. These include, among other things, encouraging people to be physically active and to create an environment that makes it easier for people to be physically active in their homes. For example, [5] use smartphone data and developed a fitness assistant framework that automatically generates a fitness schedule. The framework also incorporates social interaction to increase the engagement of its users. The most advanced state-of-the-art technology aims at serving as a substitute for a personal trainer. For instance, FitCoach [6] is a virtual fitness coach that uses wearable devices and assesses the patterns and position of its users during workouts in order to help them achieve an effective workout and to prevent them from workout injuries. Extensive experiments, both in indoor and outdoor conditions, have shown that FitCoach can assess its users’ workout and provide an adequate recommendation with accuracy >90%.

**Figure 1:**
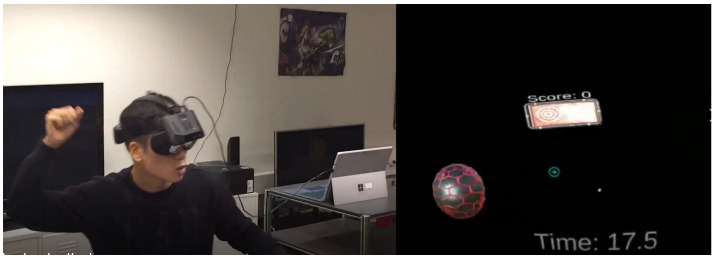
VR dodgeball game implementing ExerSense algorithm.

In our previous work, we introduced a method that provides accurate real-time segmentation, classification, and counting of both indoor and outdoor practiced physical exercises from the signal of a single inertial measurement unit (IMU) worn on the chest [7]. Targeting five types of exercises, the proposed segmentation algorithm achieved 98% precision and 94% recall, while the proposed classification method achieved 97% precision and 93% recall. We demonstrated the flexibility of proposed method by developing a virtual reality dodgeball application [8]. The application uses a wrist-mounted IMU and an HMD (head-mounted display), and it implements the ExerSense algorithm to detect a ball-throwing gesture toward the target in virtual space (Figure 1). This paper is an extension improving the motion detection method and demonstrating its robustness to various sensor wearing positions.

Recently, IMU sensors have become more widely adopted for physical activity recognition [9,10,11]. Some IMU-based systems (e.g., [9]) are used for step counting and walking detection to encourage its users to increase their ambulatory physical activity. Other methods (e.g., [10]) automatically recognize various walking workouts (e.g., walking and brisk walking). Finally, advanced IMU-based systems (e.g., [12,13]) aim at altogether bypassing the need for personal physical trainers. They monitor their users during exercise and classify their exercises technique and provide feedback to improve their workout. Compared to existing research, the proposed approach provides the three following practical enhancements. First, most existing approaches have practical limitations. For example, methods for outdoor physical activity recognition are usually based on frequency analysis, and since the number of cycles is large, a few misclassifications are tolerable, but such errors are not tolerable for plyometric exercise. The proposed method works well for short-term cyclic movement exercises (e.g., push-ups) and for long-term cyclic quick movements exercises (e.g., running and walking). Second, unlike other comparable machine-learning-based approaches that need a lot of training data, the proposed method needs one sample of motion data of each target exercises and yet performs reasonably well (accuracy >95%). Finally, although not yet validated, the proposed approach has also the potential to evaluate the quality of the workout.

## 2. Related Work

### 2.1. Behavior Recognition and Step Counting from Wearables

Step counting has been extensively studied in the ubiquitous computing community. Many works have proposed accurate algorithms to count accurately walking and running steps from a smartphone worn in the trousers pocket or at the upper arm [14,15,16], but also from a smartwatch [17,18]. Step counting is now a standard functionality in most smartphones and smartwatches. Still, false positives are still unsolved issues. The main reason for that is motion noise that produces the same signals as walking.

However, when IMUs are at the ear, they find that many of the lower-body motions are naturally “filtered out”, i.e., these noisy motions do not propagate up to the ear. Hence, the earphone IMU detects a bounce produced only from walking. Prakash et al. introduced the advantages of eSense in counting the number of steps of walking [9]. While head movement can still pollute this bouncing signal, they developed methods to alleviate the problem. Results show 95% step count accuracy even in the most difficult test case—very slow walk—where smartphone and wrist-band-type systems falter. Importantly, their system STEAR (STep counting from EARables) is robust to changes in walking patterns and scales well across different users. Additionally, they demonstrate how STEAR also brings opportunities for effective jump analysis, often crucial for exercises and injury-related rehabilitation.

Bayat et al. [19] proposed a machine-learning-based recognition system to recognize certain types of human physical activities using acceleration data generated by a user’s smartphone, and could reach an overall accuracy rate of 91%. Similarly, Balli et al. [20] can classify eight different daily human activities with high accuracy from smartwatch sensor data using a hybrid of principal component analysis and random forest algorithm. More recently, Teng et al. [21] demonstrated on several open datasets that convolutional neural network (CNN) models could improve further the performance across a variety of HAR (human activity recognition) tasks.

**Figure 2:**
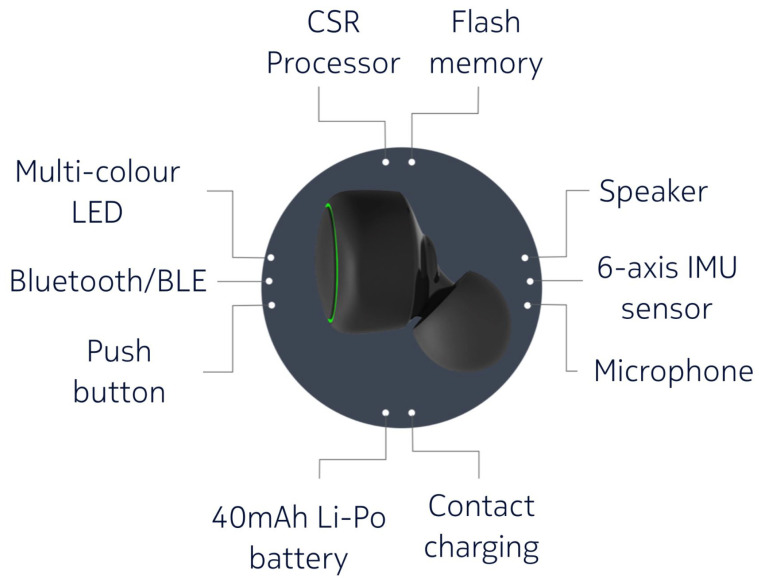
Components of eSense (quoted from [22]).

While many researchers and developers have been developing applications based on smartphones and smartwatches, Kawsar et al. [22] proposed and developed a new wearable platform called “eSense” (see Figure 2). The eSense platform consists of a pair of wireless earbuds augmented with kinetic, audio, and proximity sensing. The left earbud has a six-axis IMU with an accelerometer, a gyroscope, and a Bluetooth Low Energy (BLE) interface used to stream sensor data to a paired smartphone. Both earbuds are also equipped with microphones to record external sounds.

The use of earphones to listen to music while exercising is widespread, and though the eSense platform is still recent, it already attracted the attention of many research teams. It can simultaneously monitor behavior analyzing the sensory information and provide feedback that does not bother the visual field of the user through the acoustic interface. Indeed, repeated check of some visual feedback provided on a smartphone or smartwatch screen may be dangerous and the cause of accidents when done during exercises implying motion. For example, Prakash et al. developed an algorithm that can perform robust step counting and jump analysis from the inertial signals streamed by the eSense ear-buds [9]. In their study, they also showed the ear position is advantageous to collect motion signals since it enables to filter of lower-body noisy motions naturally. On the other hand, Radhakrishnan et al. proposed to use the eSense platform to improve user engagement during indoor weight-based gym exercises [23].

### 2.2. Vision-Based Exercise Recognition

There exist many studies that quantitatively evaluate the performance of sports and physical exercises. These researches are often based on three-dimensional (3-D) image analysis, whether it is for baseball [24,25,26,27,28,29], tennis [30,31,32,33], or games [34]. Typically, the evaluation is based on kinematics and the dynamics of joint motions of shoulder, elbow, forearm, wrist, and fingers during pitching. For example, Antón et al. [35] introduced a Kinect-based algorithm for the monitoring of physical rehabilitation exercises. The algorithm recognizes the main components of the exercises, postures, and movements in order to assess their quality of execution. Moreover, this game-like immersive framework motivates them to do the rehabilitation sessions more enjoyable. Despite only a few samples in the training step, the algorithm is capable of making real-time recognition of the exercises and achieved a monitoring accuracy of 95.16% in a real scenario when evaluated on 15 users.

In general, vision-based approaches are more accurate than wearable sensor-based approaches for exercise recognition. Although they achieve good performances, the use of a vision-based sports/exercise recognition system is limited to dedicated locations. 3-D image analysis is complex and computationally intensive. This limitation is, however, minimized by the possibility to perform some preprocessing on the sensor level.

### 2.3. Skill Science

Up to now, many researches have proposed to evaluate sports skills quantitatively. For long time, they have been principally carried out based on three-dimensional image analysis, whether it is for baseball [24,29] or tennis [30,33]. Along with the widespread use of wearable sensor devices, research and techniques for analyzing the movement of bodies and tools from acquired data are progressing in sports fields and the like by attaching sensors to the body and gears. In the field of skill science, there are some research works consisting in attaching a sensor to a tennis racket and analyze its behavior [36], and others focusing on the estimation of baseball pitching speed using a wrist-mounted acceleration sensor and laser apparatus [37]. However, most proposed accurate solutions are base on dedicated sensors (“Smart Tennis Sensor” by Sony Corporation [38]) or the wrist (“Babolat Play” by Babolat [39]), and require computer postprocessing, such that there is no real-time nor onsite feedback to improve skills.

With the popularity of smartwatches and other smart wearable devices that integrate multiple sensors, there is less need for exercise-specific hardware development. Smartwatches generally have built-in microelectromechanical systems (MEMS), IMU, and pulse rate (PR) sensors. Therefore, these devices need only software applications to be developed for each targeted sport or exercise. In their extensive review of technologies available for tennis serve evaluation, Tubez et al. raise the great prospect offered by markerless systems based on inertial measurement units for real situation evaluation [40]. Examples are the applications developed by Lopez et al. [41] for supporting an athlete or a beginner with baseball pitching action and tennis serve action. The personal sport skill improvement support application is running on Sony’s SmartWatch SWR50 and does not even need to communicate with the paired smartphone to perform onsite movement analysis and feedback. The comparative research using the proposed smartwatch applications for sport skill improvement support achieved encouraging results.

### 2.4. Recognition of Movement-Repetition-Based Exercises

One of the relevant previous work is that of Dan et al. [42], who introduced RecoFit, a system for automatically tracking repetitive exercises such as weight training and calisthenics via an arm-worn inertial sensor. They addressed three challenges: segmenting, recognizing, and counting of several repetitive exercises. They achieved precision and recall greater than 95% in segmenting exercise periods, 99%, 98%, and 96% of recognition of 4, 7, and 13 exercises, respectively, and 93% of ±1 repetition of counting accuracy. However, the method of RecoFit needs five seconds to segment and recognize exercise. In the case of a small number of counts, it cannot find correct exercise and count. It requires a dedicated device attached to the forearm; that implies a supplementary cost for users that have to buy a device for a particular and limited usage, as well as the burden of attaching a device to an unusual part of the body.

Viana et al. [43] proposed an application called GymApp, similar to the system mentioned above, but applied to workout exercise recognition. It also runs on Android OS smartwatches and monitors physical activities, for example, in fitness. It has two modes of operation: training mode and practice mode. In training mode, an athlete is advised to perform an exercise (e.g., biceps curl) with lighter weight and with the supervision of a fitness instructor to guarantee the correctness of the performed exercise. The application then gathers sensory data and builds a model for the performed exercise using supervised machine learning techniques. Then, in the practice mode, the recorded sensory data are compared with the previously acquired data. The application calculates the similarity distance and, from the result, estimates how many repetitions of the exercise were performed correctly.

More recently, Skawinski et al. [44] consider four different types of workout (pushups, situps, squats, and jumping jacks), and proposed a workout type recognition and repetition counting method based on machine learning with a convolutional neural network. Their evaluation with data from 10 subjects wearing a Movesense sensor on their chest during their workout resulted in 89.9% average detection of workout and 97.9% average detection accuracy for repetition counting.

Although the above-described studies are promising, they are based on machine learning techniques. It implies a necessary preliminary step to collect data to train a model for each type of targeted movement, as well as for each type of sensor or sensor position (wrist, chest, arm, head, etc.). This training step is a burden for the users and a disadvantage towards deploying the technology.

### 2.5. Summary

Most of the works related to detailed exercise recognition achieve around 95% for each defined exercise under the condition of only indoor workouts or only outdoor exercises like walking and running. Thus, in this research, we aim to recognize both indoor and outdoor exercises while keeping with the same accuracy. We define indoor exercises as physical activities performed on the spot, such as push-ups and sit-ups, usually performed at home or a sports gym. Contrarily, we define outdoor exercises as physical activities involving the displacement of the whole body, such as running and walking, usually performed outdoor (though you can use some running machines indoors).

Many of them are based on machine learning techniques, which often require a new dataset for each new user. Thus, this research also aims at proposing a method that provides accurate real-time segmentation, classification, and counting of physical exercises without needing recalibration for each user.

## 3. Methods

In this section, we introduce the method of the proposed system. In Section 3.1, the outline of ExerSense is presented. Then, in Section 3.2 and Section 3.3, we describe, respectively, the details of segmentation and classification. Finally, we briefly explain how counting is performed in Section 3.4.

### 3.1. Outline of ExerSense

**Figure 3:**
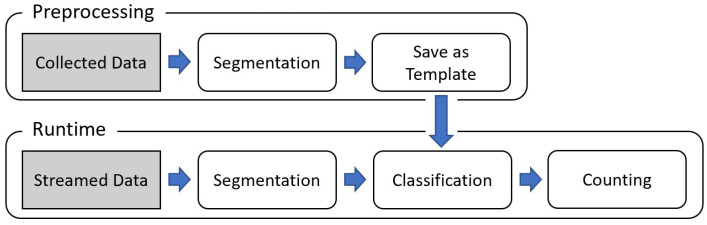
Schematic of the architecture of the proposed method.

Figure 3 represents a broad schematic of the architecture of the proposed recognition method, ExerSense. It is separated into two phases: preprocessing and runtime phase. As described later, the proposed method works independently of the kind of devices.

In the preprocessing phase, some acceleration data are collected by target devices at least one motion for each target exercise. Because the method uses a correlation-based algorithm to classify each motion, only one single motion sample of the target exercise is needed in advance. That is a significant advantage of the correlation-based approach against approaches based on machine learning. In the case of image classification, natural language processing, and so on, data are extensively available on the Internet and easy to collect physically. However, in the case of exercise recognition, it is tough to collect training data for machine learning.

The runtime phase starts with the segmentation of the streamed acceleration signal into single motions by finding the peaks in the synthetic acceleration signal. The next section explains in detail the segmentation process. Then, every segmented 3-D acceleration signal is classified by comparison with each exercise’s motion template produced in the preprocessing phase using a correlation-based algorithm, and the count of classified exercise is incremented.

### 3.2. Segmentation Algorithm for Single Motion Extraction

Hereafter we describe the process of segmentation algorithm from a 3-D acceleration signal collected at the chest during push-ups exercise. First, the synthetic acceleration of streamed inertial sensor data, which is the norm of the 3-D acceleration signal, is calculated. In the case of push-ups, peaks detection and motion segmentation may be performed using only the longitudinal acceleration of raw data. However, it is not the right solution since this research targets not only push-ups but also other types of exercise, including those that do not imply movements in the longitudinal direction. Therefore, the synthetic acceleration is more appropriate, though it presents a disadvantage of reducing the differences between movements that are similar but along a different axis.

The result of the norm includes much noise. Applying short-term energy enables not only to emphasize significant signal variations but also to smooth them. Smoothing is important to detect only motion start and end peaks easily.

Then, we used a sliding window of 0.25 s length to detect peaks. The tempo of the running steps is the shortest tempo among regular exercises. After observing various persons running, the fastest tempo more than three but less than four steps per second. Hence, to avoid having two steps in a sliding window, we chose 0.25 s as the optimal size. If the center value of the window is the maximum value of the window, then it is determined as a peak. The fourth plot shows detected peaks plotted on the smoothed norm of acceleration signal collected during push-ups exercise.

Finally, the synthetic acceleration signal (*x* × *x* + *y* × *y* + *z* × *z*) is segmented by extracting the data between the period of two consecutive peaks. Such, we define a “segment of exercise” as the raw acceleration data between the time interval of two consecutive peaks extracted from the smoothed synthetic acceleration signal, and containing a single motion of an exercise (e.g., one step, one jump, one push-up, etc.).

**Figure 4:**
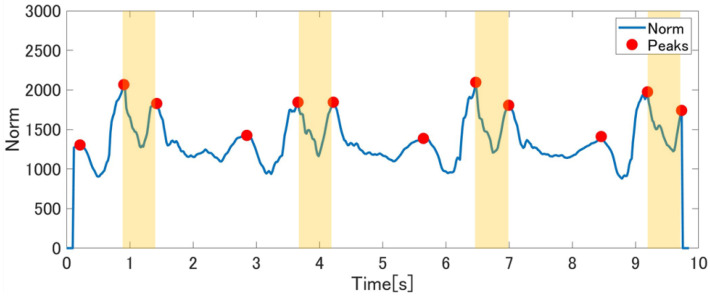
The smoothed synthetic acceleration signal and detected peaks during sit-ups exercise.

In most cases, one peak is detected for each motion. However, in the case of sit-ups, multiple peaks are detected for each motion (see Figure 4). To be able to deal with this case, one of the peak-to-peak periods (yellow-colored in Figure 4) is defined as sit-up base motion. Yellow-colored peak-to-peak represents “wake-up” motion during sit-up. Because “wake-up” is the most important movement for sit-up training, we selected the area.

### 3.3. Classification of Extracted Motion Segments

**Figure 5:**
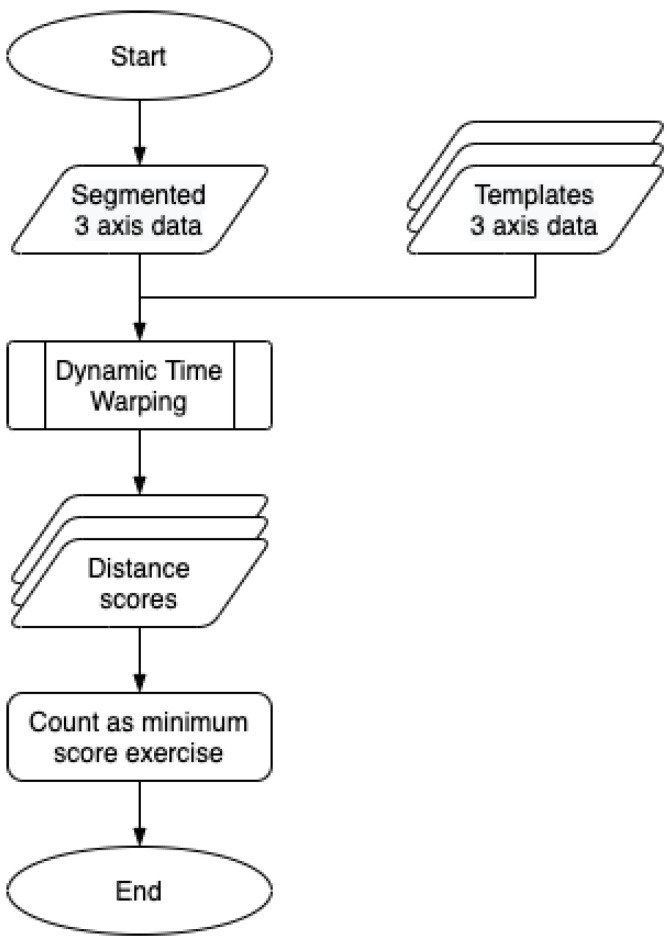
Flow chart of the processing flow of the proposed method to classification extracted motion signal segments.

Figure 5 shows the processing flow of the proposed classification method. After extracting the 3-D acceleration signal corresponding to a single motion through the segmentation process, the dynamic time warping (Algorithm 1) algorithm is applied to calculate the distance between every template signal and the extracted signals. The dynamic time warping (DTW) can calculate the distance between two time series data that have different lengths. This is a crucial property since it offers the capability to deal with the shape of signals issued from one identical exercise, independently of the speed the exercise motion is performed. Finally, the proposed method classifies the exercise that has the minimum DTW score as the ongoing exercise.

In our previous work [7], artificial coefficients are applied to DTW score to increase the performance. These coefficients were determined by variances of the three axes that are affected by the body influence, the direction of maximum movement, and the intensity of movement. However, these coefficients were predefined by authors based on experiences and only for the chest-mounted sensor. In this work, we removed the coefficients to compare multiple device positionings.
**Algorithm 1** Dynamic Time WarpingDTW⇐array[0…n,0…m]**for**i=0…n**do** DTW[i,0]⇐infinity
**end for****for**j=0…m**do** DTW[0,j]⇐infinity
**end for**DTW[0,0]⇐0**for**i=0…n**do** **for** j=0…m **do**
  cost⇐||(s[i]−t[j])||
  DTW[i,j]⇐cost+min(DTW[i−1,j],DTW[i,j−1],DTW[i−1,j−1])
 **end for**
**end for****return**DTW[n,m]

### 3.4. Counting

After the classification step, it is easy to count each exercise. Only what we need to do is to iterate by one the counter for each classified exercise. However, in the case of sit-ups, the proposed method divides one motion into three segments. One of the three segments will be similar to template data, but other similarities are unlikely. Thus, we can count correctly with the combinations of segmentation and classification.

## 4. Experimental Results

This section presents the experimental datasets in Section 4.1 and describes the proposed method’s accuracy in Section 4.2.

### 4.1. Datasets

The experimental conditions are described in Section 4.1.1, the targeted exercises are defined in Section 4.1.2, and the collected segments are discussed in Section 4.1.3.

#### 4.1.1. Conditions

**Figure 6:**
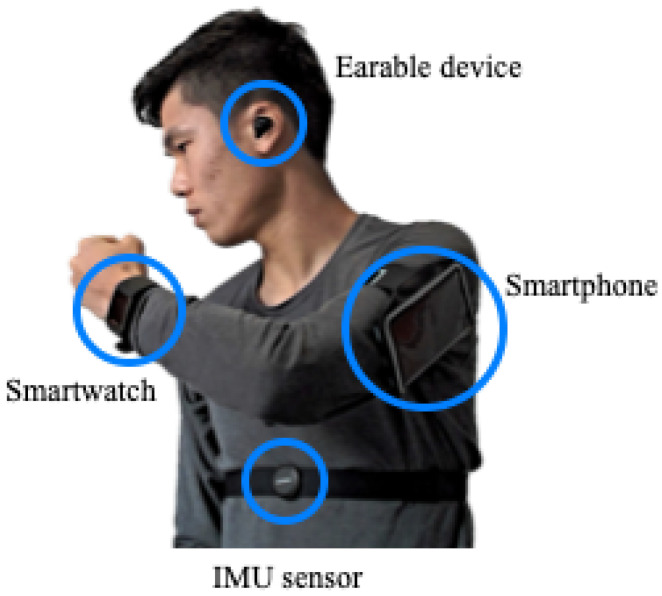
Sensors used for the experiment and their positions.

- **Experimental circuit** —As mentioned in the introduction, this research targets exercises including indoor workouts and outdoor activities. A circuit to perform five exercises has been created to evaluate the proposed method. The order of the five exercises, which is explained in the next section, is determined randomly and systematically.
- **Participants** —Fifteen university student participants were recruited. Participants varied in weight from 58 kg to 80 kg, and self-assessed as performing exercise “at least once a week,” with an average of four times a week. Each participant performed all exercises once according to the conditions described above. Due to the missing value of three participants, we used valid data from 12 participants to validate the proposed method.
- **Sensors** —This research aims to develop an exercise recognition and counting method that is deployable with various commercially available general use wearable devices (e.g., smartwatches, smart glasses, chest bands, etc.). Such a method needs to be robust to devices and their positioning. One can assume that the chest movement, like the head, has less noise than other body parts. Chest sensors are also commonly used by people practicing exercise several times a week to monitor their heart rate. Hence, our previous study [7] demonstrated the validity of the proposed method based on the signal of an IMU mounted at the chest. As a chest-mounted sensor, we used Suunto Movesense Sensor HR+ (Movesense), consisting of a nine-axis motion sensor, heart rate sensor, and Bluetooth within 10 g [45]. In this work, in addition to Movesense mounted at the chest, we used three other wearable devices that are often worn by people when practicing physical exercises: a smartwatch attached to the left wrist, a smartphone attached to the upper left arm, and a wearable device (Nokia Bell Labs eSense) attached to the left ear (see Figure 6). All four wearable devices integrate a nine-axis IMU. The smartwatch and smartphone have some storage so that they could collect data by themselves. The chest-mounted and ear-mounted wearable devices do not have storage, so these two were connected to a smartphone by Bluetooth and streamed the acceleration data.

#### 4.1.2. Definition of Exercises

The proposed method was evaluated and validated on the following five exercises.

1. Running (right/left)
2. Walking (right/left)
3. Jumping
4. Push-up
5. Sit-up

**Figure 7:**
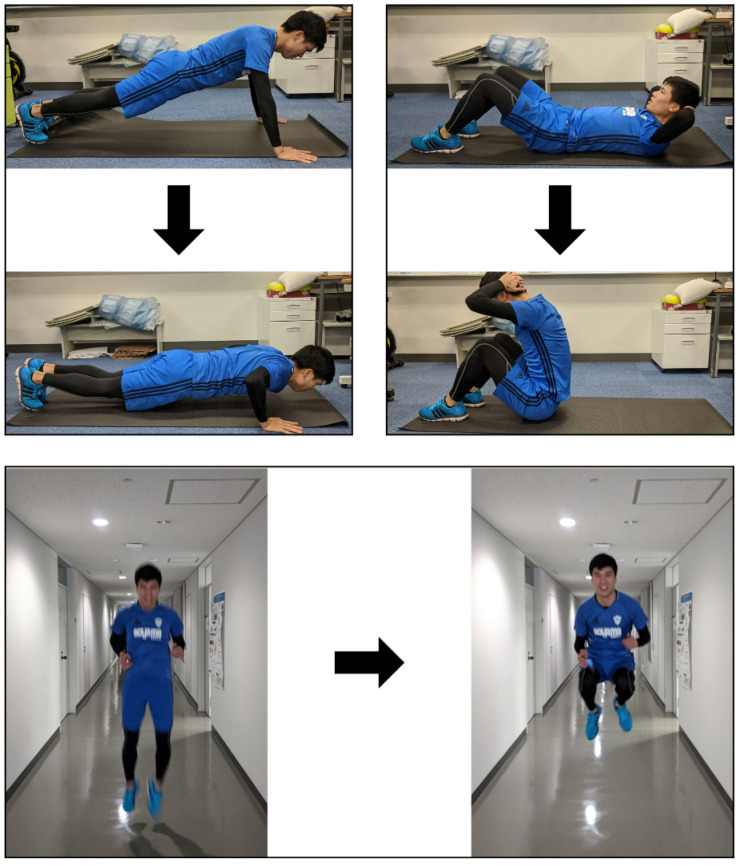
Pictures used to define push-ups (**top left**), sit-ups (**top right**), and jump (**bottom**) for subjects.

The reason why we chose these five exercises is that we suppose that exercise consists of indoor workouts and outdoor running/walking. Additionally, these five exercises can be completed on flat ground without any equipment. Participants ran and walked more than 20 steps each without caring whether they start with the right or left foot. They performed jumps, push-ups, and sit-ups around ten times each. The movements of jumping, push-ups, and sit-ups were predefined and explained using demonstration photos because there are various kinds of movements (see Figure 7).

In the case of the ear and the chest, it does not matter whether the running or the walking step is taken by the right foot or left foot. On the contrary, the upper arm and the wrist movements are different between the the right step and the left step. Accordingly, we separated the templates of the upper arm and the wrist by right and left. While there are five ear and chest templates, there are seven upper arm and wrist templates.

In previous work [7], the author performed the exercises to produce the templates for all five exercises, which are necessary for real-time classification. This time, we chose the templates randomly from the participants data and calculate the classification accuracy excluding the templates. Additionally, we repeated the validating process 50 times and got the mean accuracy.

#### 4.1.3. Number of Segments

Under the conditions mentioned above, we collected segments of five for each of the exercises using four different sensors. Table 1 shows the number of collected segments. Although some sensors have several missing values, we generally used the number of segments for validation.

### 4.2. Performances

The recall of segmentation and the performances metrics of classification are described in Section 4.2.1 and Section 4.2.2, respectively.

#### 4.2.1. Recall of Segmentation

We counted the segments cut out accurately by the proposed algorithm. Table 2 shows the recall of segmentation against truth counts.

#### 4.2.2. Performance of Classification

From the all collected segments, we randomly chose the template segments for each exercises and classified other segments. Additionally, we repeated the random validation process 50 times to avoid redundancy. Table 3 shows the classification accuracy for all five exercises listed by sensor position. As shown in Table 3, the chest was the most accurate position (97.2%) with a minimal standard deviation (4.4%), as expected in our previous work [7]. Next came the upper arm and the wrist (93.1% and 83.5%), with relatively low standard deviations (3.1%, 5.6%). The ear was the less accurate position with an average accuracy of 78.4% and a large standard deviation of 10%. Regarding the classification performances per exercise, jumping and push-ups had the worst F1 value that tended to have a large standard deviation, especially with the earable device, prone to be loosely attached. We can also raise the point that using a wrist-worn device, the lowest F1 value was for push-ups (74.5% ± 14.4) due to little motion of the wrist during push-ups.

### 4.3. Comparison with Machine Learning Method

The proposed method extracts one sample of motion data of each target exercise from one subject data and uses it to recognize exercise data collected from unknown users. To compare the accuracy with a conventional machine learning method, we used both leave-one-subject-out and leave-other-subjects-out cross-validation. Indeed, leave-one-subject-out cross-validation uses plural subjects data for training, while the proposed method used only one subject data as a reference. In leave-other-subjects-out cross-validation, the model training is performed with only one subject’s data and testing with all others. We repeated both validation methods for each user (in or out) and calculated the average confusion matrix for linear support vector machine (SVM) (see Table 4 and Table 5).

Compared to the proposed method, for most types of exercise and sensor position, the machine learning method gives better accuracy when trained with plural users (leave-one-out) but lower accuracy when trained with only one user. These results confirm that the proposed method is advantageous compared to conventional machine learning methods when retraining for each new user is not affordable.

## 5. Discussion

### 5.1. Discussion about the Segmentation

As shown in Table 2, the chest-mounted IMU, arm-mounted smartphone, and wrist-mounted smartwatch achieved 91% recall. Even the worst one, the ear-mounted device, achieved 84% recall. It is said that the proposed segmentation algorithm works well at various positions.

**Figure 8:**
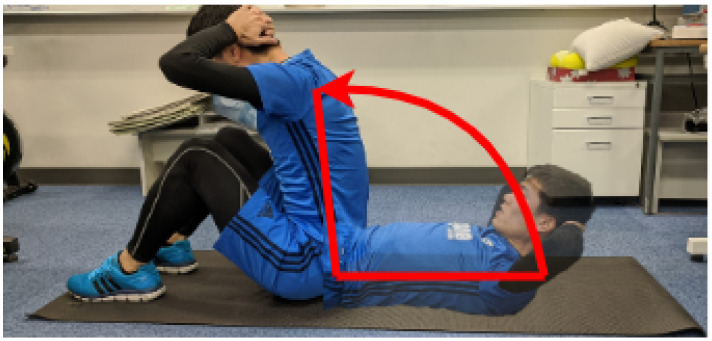
The change of the most moving axis during one sit-up.

However, we can see the significant differences for each exercise. While walking and push-ups achieved more than 90% recall, sit-ups achieved only 52–61%. The reason why the proposed segmentation method overlooked many sit-ups segments is that the most change in the moving axes occurs during one motion. As showing in Figure 8, the sit-up motion is circular, and it causes the most change in the moving axes. As a result, when the norm of three axes was calculated, plural peaks appeared. These peaks are ignored at the step of smoothing if they are small. However, in some cases, the invalid peaks are big and remain so after smoothing. Then, it is detected as the cutting point of segments.

### 5.2. Discussion about the Classification

Table 6 shows that the range of length of collected exercise segments for each device (position) and exercise type has significant variations. It means that each exercise is performed at various speeds. The DTW (dynamic time warping) algorithm has the specificity to be robust to different data lengths such that the proposed method was not affected by the same exercise’s different execution speeds.

**Figure 9:**
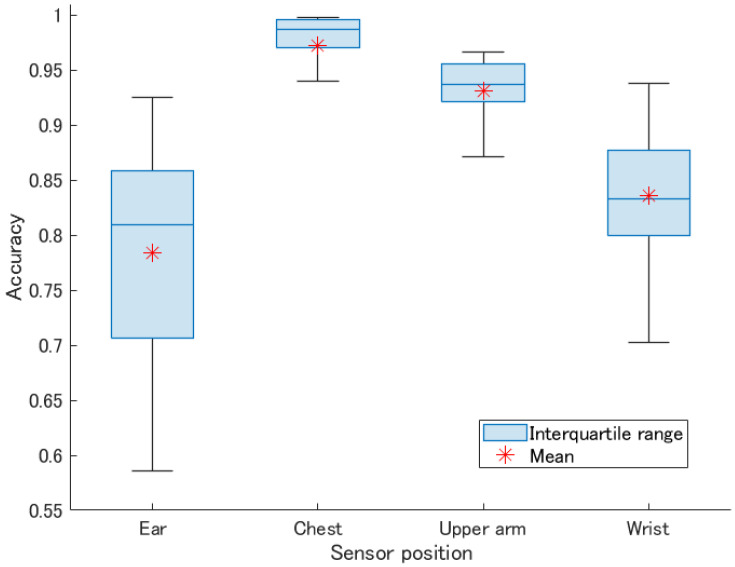
Box plot of the accuracy of each random validation (*n* = 50).

Figure 9 illustrates the box plot of 50 times repeated validation using random exercise segment template selection, with a mark of the mean accuracy for each exercise. Though the median and quarter percentiles limits for ear and wrist positions are partially overlapping, all device positions’ mean accuracies are significantly different at the 5% significance level as summed up in Table 7. As described in Section 4.2.2, the ear mounted sensor’s average classification accuracy was significantly lower than others. The head movements are less restricted and more prone to noisy motions than trunk and hand movements during physical exercises. Hence, the accuracy is more affected by the quality of the selected exercise template. The large standard deviation also confirms this issue.

However, since the proposed method is based on one template segment per exercise and position, this result also shows the importance of the template exercise segment’s quality. Considering this, we should also refer to the maximum accuracy to fairly evaluate the potential of the proposed approach. Indeed, the maximum accuracy is the accuracy obtained when selecting optimal template exercise segments. In that case, the classification accuracy is 99.8%, 97.1%, 94.2%, and 93.4% for the chest, the upper arm, the wrist, and the ear, respectively (see Table 8). While the proposed method uses only one exercise segment template to recognize unknown users’ exercises, such performances are equivalent to the machine learning model evaluated by leave-one-out cross-validation. Hence, using optimal template exercise segments, the proposed method is robust to various wearable device positions.

## 6. Conclusions

In this research, we proposed ExerSense, a method to segment, classify, and count multiple physical exercises in real time. ExerSense is based on the correlation method because only one motion is needed in advance. In the case that is difficult to collect data for a physical exercise, it is more advantageous to use the correlation method instead of the machine learning method.

We collected acceleration data of five exercises by four different positioned sensors. In order to validate our proposed segmentation method, we counted the correct extracted segments. It recalled more than 91% of segments, except 84% of the ear. Using the accurately extracted segments, we validated the classification method. The most accurate one was the Movesense sensor mounted to the chest; it achieved 99% accuracy. The smartphone mounted to the upper arm was a close second with 94%. The smartwatch mounted to the wrist was third with 86%, and the worst one among the four was the eSense mounted to the ear with 76%.

The proposed method, ExerSense, segments and classifies multiple exercises accurately in general usage devices. We found that the ExerSense system works at various positions. Though it has room for improvement of sit-up segmentation, it achieved high accuracy for most of regular exercises. In future works, we will improve the ExerSense algorithm and test it on other exercises and other positions.

## Figures and Tables

**Table 1:** Number of collected segments.

Running	Walking	Jumping	Push-Ups	Sit-Ups	Total
327	396	123	125	120	1092

**Table 2:** Segmentation recalls.

		Each Exercise					Total
		Running	Walking	Jumping	Push-Ups	Sit-Ups	
Position	Ear	0.859	0.909	0.724	0.920	0.600	0.841
	Chest	0.970	0.961	0.965	0.943	0.537	0.914
	Upper arm	0.972	0.977	0.951	0.936	0.517	0.918
	Wrist	0.982	0.949	0.894	0.904	0.610	0.916

**Table 3:** Classification performance of randomly selected template exercise segments (50 iterations).

		F1 Value (SD: Standard Deviation) of Each Exercise					Mean Accuracy
		Running	Walking	Jumping	Push-Ups	Sit-Ups	
Position	Ear	0.757	0.803	0.696	0.700	0.880	0.784
		(0.178)	(0.156)	(0.238)	(0.218)	(0.112)	(0.100)
	Chest	0.952	0.988	0.905	1.000	0.991	0.972
		(0.111)	(0.035)	(0.196)	(0.001)	(0.006)	(0.044)
	Upper arm	0.914	0.964	0.937	0.882	0.853	0.931
		(0.039)	(0.021)	(0.090)	(0.100)	(0.185)	(0.031)
	Wrist	0.809	0.878	0.778	0.745	0.915	0.835
		(0.096)	(0.054)	(0.151)	(0.144)	(0.131)	(0.056)

**Table 4:** Leave-one-subject-out cross-validation accuracy of conventional machine learning methods (linear SVM model).

		Each Exercise					Total
		Running	Walking	Jumping	Push-Ups	Sit-Ups	
Position	Ear	0.964	0.990	0.934	0.864	0.934	0.957
	Chest	0.988	0.992	0.991	1.000	0.991	0.990
	Upper arm	0.984	0.965	0.995	0.995	0.804	0.952
	Wrist	0.976	0.965	0.995	0.932	0.952	0.944

**Table 5:** Leave-other-subjects-out cross-validation accuracy of conventional machine learning methods (linear SVM model).

		Each Exercise					Total
		Running	Walking	Jumping	Push-Ups	Sit-Ups	
Position	Ear	0.848	0.937	0.832	0.685	0.620	0.771
	Chest	0.911	0.949	0.868	0.989	0.919	0.869
	Upper arm	0.936	0.968	0.903	0.941	0.759	0.835
	Wrist	0.890	0.938	0.877	0.847	0.639	0.795

**Table 6:** Range (min-max) of exercise segment length in milliseconds for each exercise and device.

	Run	Walk	Jump	Push-Up	Sit-Up
Ear	96–654	269–846	173–1019	308–1250	404–1308
Chest	250–500	365–769	577–827	558–1673	462–1250
Upper arm	232–532	305–749	586–862	596–1680	601–1217
Wrist	179–623	226–981	566–840	245–783	377–1236

**Table 7:** *p*-values of accuracy between two positions.

Position 1	Position 2	Mean Diff	*p*-Value
Ear	Chest	−0.188	0.000
Ear	Upper arm	−0.147	0.000
Ear	Wrist	−0.051	0.000
Chest	Upper arm	0.041	0.007
Chest	Wrist	0.137	0.000
Upper arm	Wrist	0.096	0.000

**Table 8:** Maximum and minimum classification accuracy of 50 random template exercise segments.

Position	Mean	Max	Min	SD
Ear	0.784	0.925	0.586	0.100
Chest	0.972	0.998	0.737	0.044
Upper arm	0.931	0.967	0.838	0.031
Wrist	0.835	0.938	0.703	0.056

## Data Availability

Data collected or analyzed in this study are not available for sharing.

## References

[1] Dahn J.R., And F.J.P. (2005). Exercise and well-being: A review of mental and physical health benefits associated with physical activity. Curr. Opin. Psychiatry.

[2] Warburton D.E. (2006). Health benefits of physical activity: The evidence. Can. Med. Assoc. J..

[3] Dishman R.K., Sallis J.F., Orenstein D.R. (1985). The determinants of physical activity and exercise. Public Health Rep..

[4] Tuso P. (2015). Strategies to Increase Physical Activity. Perm. J..

[5] Dharia S., Eirinaki M., Jain V., Patel J., Varlamis I., Vora J., Yamauchi R. (2018). Social recommendations for personalized fitness assistance. Pers. Ubiquitous Comput..

[6] Guo X., Liu J., Chen Y. (2020). When your wearables become your fitness mate. Smart Health.

[7] Ishii S., Nkurikiyeyezu K., Luimula M., Yokokubo A., Lopez G. (2020). ExerSense: Real-Time Physical Exercise Segmentation, Classification, and Counting Algorithm Using an IMU Sensor. Proceedings of the International Conference on Activity and Behavior Computing (ABC).

[8] Ishii S., Luimula M., Yokokubo A., Lopez G. VR Dodge-ball: Application of Real-time Gesture Detection from Wearables to ExerGaming. Proceedings of the 2020 11th IEEE International Conference on Cognitive Infocommunications (CogInfoCom).

[9] Prakash J., Yang Z., Wei Y.L., Choudhury R.R. (2019). STEAR: Robust Step Counting from Earables. Proceedings of the 1st International Workshop on Earable Computing (EarComp’19).

[10] Wahjudi F., Lin F.J. IMU-Based walking workouts recognition. Proceedings of the IEEE 5th World Forum on Internet of Things, WF-IoT 2019—Conference Proceedings.

[11] Hausberger P., Fernbach A., Kastner W. IMU-based smart fitness devices for weight training. Proceedings of the IECON Proceedings (Industrial Electronics Conference).

[12] O’reilly M.A., Slevin P., Ward T., Caulfield B. (2018). A wearable sensor-based exercise biofeedback system: Mixed methods evaluation of formulift. JMIR MHealth UHealth.

[13] Crema C., Depari A., Flammini A., Sisinni E., Haslwanter T., Salzmann S. IMU-based solution for automatic detection and classification of exercises in the fitness scenario. Proceedings of the SAS 2017 IEEE Sensors Applications Symposium.

[14] Brajdic A., Harle R. (2013). Walk Detection and Step Counting on Unconstrained Smartphones. Proceedings of the 2013 ACM International Joint Conference on Pervasive and Ubiquitous Computing (UbiComp ’13).

[15] Salvi D., Velardo C., Brynes J., Tarassenko L. An Optimised Algorithm for Accurate Steps Counting From Smart-Phone Accelerometry. Proceedings of the 2018 40th Annual International Conference of the IEEE Engineering in Medicine and Biology Society (EMBC).

[16] Casado F.E., Rodríguez G., Iglesias R., Regueiro C.V., Barro S., Canedo-Rodríguez A. (2020). Walking Recognition in Mobile Devices. Sensors.

[17] Genovese V., Mannini A., Sabatini A.M. (2017). A Smartwatch Step Counter for Slow and Intermittent Ambulation. IEEE Access.

[18] Brondin A., Nordström M., Olsson C.M., Salvi D. (2020). Open Source Step Counter Algorithm for Wearable Devices. 10th International Conference on the Internet of Things Companion.

[19] Bayat A., Pomplun M., Tran D.A. (2014). A Study on Human Activity Recognition Using Accelerometer Data from Smartphones. Procedia Comput. Sci..

[20] Balli S., Sağbaş E.A., Peker M. (2019). Human activity recognition from smart watch sensor data using a hybrid of principal component analysis and random forest algorithm. Meas. Control.

[21] Teng Q., Wang K., Zhang L., He J. (2020). The Layer-Wise Training Convolutional Neural Networks Using Local Loss for Sensor-Based Human Activity Recognition. IEEE Sensors J..

[22] Kawsar F., Min C., Mathur A., Montanari A. (2018). Earables for personal-scale behavior analytics. IEEE Pervasive Comput..

[23] Radhakrishnan M., Misra A. Can earables support effective user engagement during weight-based gym exercises?. Proceedings of the 2019 ACM International Joint Conference on Pervasive and Ubiquitous Computing.

[24] Feltner M., Dapena J. (1986). Dynamics of the shoulder and elbow joints of the throwing arm during a baseball pitch. J. Appl. Biomech..

[25] Dillman C.J., Fleisig G.S., Andrews J.R. (1993). Biomechanics of pitching with emphasis upon shoulder kinematics. J. Orthop. Sport. Phys. Ther..

[26] Werner S.L., Fleisig G.S., Dillman C.J., Andrews J.R. (1993). Biomechanics of the elbow during baseball pitching. J. Orthop. Sport. Phys. Ther..

[27] Wang Y.T., Ford H., Ford H., Shin D.M. (1995). Three-dimensional kinematic analysis of baseball pitching in acceleration phase. Percept. Mot. Skills.

[28] Barrentine S.W., Matsuo T., Escamilla R.F., Fleisig G.S., Andrews J.R. (1998). Kinematic analysis of the wrist and forearm during baseball pitching. J. Appl. Biomech..

[29] Tomoyuki M., Yoshihiro T., Tsuyoshi M. (2000). Biomechanical characteristics of sidearm and underhand baseball pitching: Comparison with those of overhand and three-quarter-hand pitching. Jpn. J. Biomech. Sport. Exerc..

[30] Emmen H., Wesseling L., Bootsma R., Whiting H., Van Wieringen P. (1985). The effect of video-modelling and video-feedback on the learning of the tennis service by novices. J. Sport. Sci..

[31] Chow J., Carlton L., Lim Y.T., Chae W.S., Shim J.H., Kuenstar A., Kokubun K. (2003). Comparing the pre-and post-impact ball and racquet kinematics of elite tennis players’ first and second serves: A preliminary study. J. Sport. Sci..

[32] Chiang C., Nien Y., Chiang J.Y., Shiang T. (2007). Kinematic analysis of upper extremity in tennis flat and topspin serve. J. Biomech..

[33] Razali R., Suwarganda E., Zawaki I. The effect of direct video feedback on performance of tennis serve. Proceedings of the ISBS-Conference Proceedings Archive.

[34] Nakai A., Pyae A., Luimula M., Hongo S., Vuola H., Smed J. (2015). Investigating the effects of motion-based Kinect game system on user cognition. J. Multimodal User Interfaces.

[35] Antón D., Goni A., Illarramendi A. (2015). Exercise recognition for Kinect-based telerehabilitation. Methods Inf. Med..

[36] Masuda D., Tasaka K., Ohgishi T., Obana S. Proposal of Progress Assist System for Tennis using Wearable Sensors. Proceedings of the Multimedia, Distributed, Cooperative and Mobile Symposium (DICOMO’2014).

[37] Saitou K., Ohgi Y., Inoue S., Ichikawa H., Yamagishi M., Miyaji C., Takai S. (2002). Estimation of Ball Speed in Baseball Pitching from Acceleration Measured at the Wrist. Jpn. J. Phys. Educ. Hlth. Sport Sci..

[38] SONY Corp, Smart Tennis Sensor. http://smartsports.sony.net/tennis/JP/ja/.

[39] Babolat, Babolat Play. http://ja.babolatplay.com/play.

[40] Tubez F., Schwartz C., Paulus J., Croisier J.L., Brüls O., Denoël V., Forthomme B. (2018). Which tool for a tennis serve evaluation? A review. Int. J. Perform. Anal. Sport.

[41] Lopez G., Abe S., Hashimoto K., Yokokubo A. On-site personal sport skill improvement support using only a smartwatch. Proceedings of the 2019 IEEE International Conference on Pervasive Computing and Communications Workshops (PerCom Workshops).

[42] Morris D., Saponas T.S., Guillory A., Kelner I. RecoFit: Using a wearable sensor to find, recognize, and count repetitive exercises. Proceedings of the SIGCHI Conference on Human Factors in Computing Systems.

[43] Viana P., Ferreira T., Castro L., Soares M., Pinto J.P., Andrade T., Carvalho P. GymApp: A Real Time Physical Activity Trainner on Wearable Devices. Proceedings of the 2018 11th International Conference on Human System Interaction (HSI).

[44] Skawinski K., Montraveta R.F., Findling R., Sigg S. (2019). Workout Type Recognition and Repetition Counting with CNNs from 3D Acceleration Sensed on the Chest. Advances in Computational Intelligence. IWANN 2019. Lecture Notes in Computer Science.

[45] Suunto Movesense Sensor HR+. https://www.movesense.com/product/movesense-sensor-hr/.

